# Small Cell Transformation of Metachronous ALK + ve Lung and Prostatic Adenocarcinoma following Initial Response to Ceritinib and Androgen Deprivation Therapies and Metastases to Two Different Organs: Guidance through FDG-PET/CT and ^68^ Ga-PSMA-11 PET–CT and Biopsy–Immunohistochemistry Correlation

**DOI:** 10.1055/s-0045-1813675

**Published:** 2025-11-23

**Authors:** Rangat Bagasariya, Keerti Sitani, Trupti Pai, Sandip Basu

**Affiliations:** 1 Radiation Medicine Centre, Bhabha Atomic Research Centre, Tata Memorial Hospital Annexe, Mumbai, Maharashtra, India; 2 Homi Bhabha National Institute, Mumbai, Maharashtra, India; 3 Department of Pathology, Tata Memorial Hospital, Mumbai, Maharashtra, India

**Keywords:** prostatic adenocarcinoma, lung adenocarcinoma, ALK-positive lung adenocarcinoma, small cell transformation, ^18^ F-FDG-PET/CT, ^68^ Ga-PSMA-11 PET/CT

## Abstract

Metachronous malignancies carry poor prognosis and pose certain challenges for management of two different malignancies simultaneously. A 59-year-old male, a patient of ALK+ lung adenocarcinoma demonstrating excellent initial response to targeted therapy with ALK inhibitor ceritinib, developed prostatic adenocarcinoma with neuroendocrine differentiation 36 months later, which was treated with abiraterone and leuprolide. Dual-tracer positron emission tomography (PET) imaging with ^18^ F-FDG-PET/computed tomography (CT) and ^68^ Ga-PSMA-11 PET/CT showed complete response of prostatic adenocarcinoma with controlled serum prostate-specific antigen level, showed new-onset metastatic brain and liver lesions 12 months later, the biopsy of the later revealed metastatic small cell neoplasia. The clinical profile, dual tracer PET/CT and immunohistochemistry correlation assisted in concluding that prostate adenocarcinoma had transformed into small cell type, which metastasized to liver, whereas the lung adenocarcinoma had metastasized to brain.

## Introduction

When two distinct primary malignancies are diagnosed more than 6 months apart, they are classified as metachronous malignancies with reported prevalence of 0.73 to 11.7%. 1 Possible risk factors include genetics, viral infection, smoking, addiction, environmental, chemotherapy, or radiation.

Anaplastic lymphoma kinase (ALK)-positive lung adenocarcinoma is an uncommon entity and represents 4 to 5% of all nonsmall cell lung carcinoma (NSCLC) cases. 2 These cases present at younger age, has male preponderance, and is frequent in those with light smoking history of <10 pack years. 3 ALK inhibitors have revolutionized the concept of targeted therapies in ALK-positive lung cancers, first generation involving, crizotinib, second generation alectinib, ceritinib, brigatinib, and third generation lorlatinib. 4 5 6

Prostate adenocarcinoma is the most common malignancy in men and second most common cause of cancer-related deaths. Prostatic adenocarcinomas with scattered foci of neuroendocrine immunohistochemical expression are called prostatic adenocarcinoma with neuroendocrine differentiation. The incidence in primary prostate cancers is approximately 1%, whereas in metastatic castration-resistant prostate cancer it is up to 25 to 30%. 7 Prostate cancers follow a graded treatment regimen. Localized prostate cancer (T1–T2, N0, M0) is treated with active surveillance using digital rectal examination, serum prostate-specific antigen (PSA), magnetic resonance imaging, and biopsy. Also, radical prostatectomy and if needed EBRT. 8 Locally advanced prostate cancers (T3–T4 or N + ) is treated with androgen deprivation therapy and radiotherapy. 9 Metastatic hormone sensitive prostate carcinoma is best treated with androgen deprivation therapy and orchidectomy. 10 Nonmetastatic castration-resistant prostate cancers are treated using enzalutamide. Most important applications of nuclear medicine and molecular imaging come in metastatic castration-resistant prostate carcinomas, where the most preferred treatment modalities include abiraterone (CYP inhibitors), enzalutamide (AR inhibitors), docetaxel, cabazitaxel (first- and second-line chemotherapies), radionuclide therapy using ^177^ Lu PSMA and ^225^ Ac PSMA. 11 12

## Case Report

**Fig. 1:**
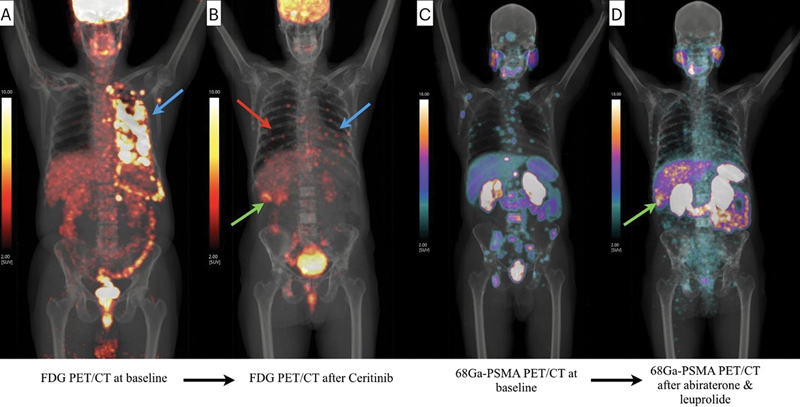
^18^ F-FDG-PET/CT MIP image at baseline ( **A** ) and after ceritinib treatment ( **B** ) showing significant reduction of FDG avid disease bulk of left lung (blue arrows) with new-onset lung nodules in right lung (red arrow in **B** ) and a liver segment VI lesion (green arrow in **B** ). ^68^ Ga-PSMA-11 PET/CT MIP image at baseline ( **C** ) and after androgen deprivation therapy ( **D** ) showing excellent response of all the metastatic skeletal lesions but a new-onset metastatic liver lesion (green arrow in **D** ). CT, computed tomography; MIP, maximum intensity projection; PET, positron emission tomography.

A 59-year-old male presented with dyspnea and chest pain. CT scan revealed left pleural thickening with multiple subpleural nodules and bilateral parenchymal lung nodules, which raised suspicion of metastatic lung carcinoma. Biopsy of left lung lower lobe lesion suggested adenocarcinoma. Immunohistochemistry showed TTF-1 (thyroid transcription factor-1)-positive, EGFR negative, and ALK-positive results. Baseline ^18^ F-FDG-PET/CT was done, which revealed FDG avid left lung pleural and bilateral parenchymal lung nodules and FDG avid axillary, mediastinal, and abdominal metastatic lymphadenopathy ( Fig. 1A ). Targeted therapy with ALK inhibitor ceritinib was initiated thereafter.

Thirty-six months later, while still on ceritinib, the patient developed symptoms of urgency and incomplete bladder voiding. Ultrasonography showed gross prostatomegaly. Serum PSA was elevated (158.75 ng/mL). Biopsy revealed conventional prostatic adenocarcinoma with neuroendocrine differentiation, Gleason's score 5 + 5 = 10, Grade group V. Immunohistochemistry was NKX3.1 and PSA-positive, synaptophysin weakly positive, chromogranin, and TTF-1-negative. Baseline ^68^ Ga-PSMA-11 PET/CT ( Fig. 1C ), which was undertaken 36 months after the baseline FDG-PET/CT scan, showed PSMA expressing prostate mass with bladder wall and seminal vesicles involvement, PSMA expressing metastatic pelvic, abdominal, mediastinal, and cervical lymphadenopathy and widespread sclerotic skeletal metastases ( Fig. 1C ). Androgen deprivation therapy was initiated with abiraterone and leuprolide.

**Fig. 2:**
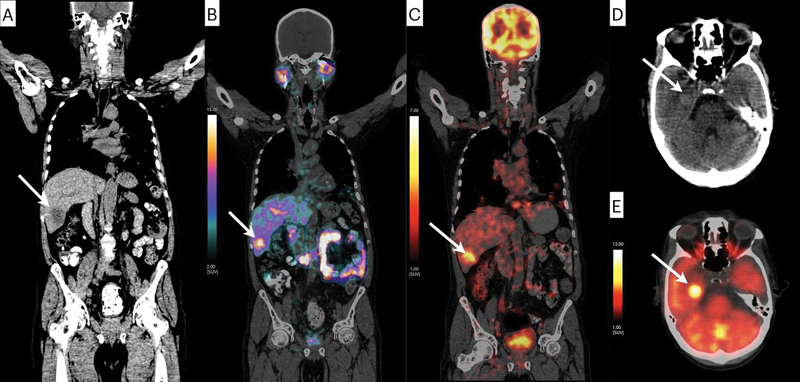
Coronal noncontrast CT ( **A** ), fused ^68^ Ga-PSMA-PET/CT ( **B** ), and fused ^18^ F-FDG-PET/CT ( **C** ) showing a new-onset metastatic lesion of liver segment VI. Transaxial noncontrast CT ( **D** ) and fused ^18^ F-FDG-PET/CT ( **E** ) showing a metastatic lesion in right temporal lobe. CT, computed tomography; PET, positron emission tomography.

**Fig. 3:**
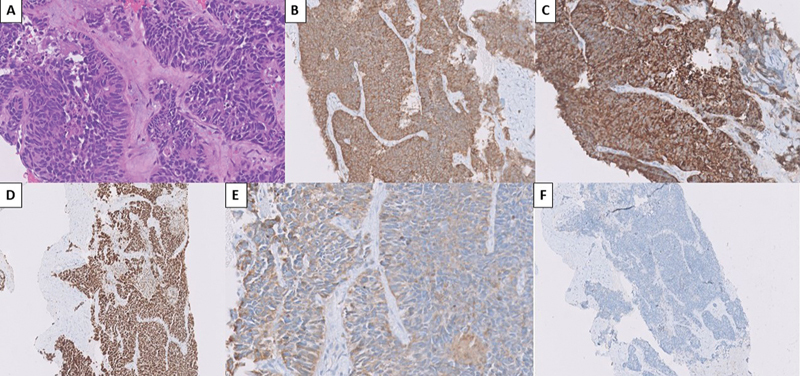
Histopathology and immunohistochemistry of the new-onset metastatic liver lesion. ( **A** ) Hematoxylin and eosin staining showing small cell carcinoma. Immunohistochemistry markers showing ( **B** ) synaptophysin-positive, ( **C** ) chromogranin-positive, ( **D** ) TTF-1-positive, ( **E** ) AE1/AE3-positive, ( **F** ) NKX3.1 negative staining.

Forty-five months after lung adenocarcinoma and 12 months after prostatic adenocarcinoma diagnoses, ^18^ F-FDG-PET/CT and ^68^ Ga-PSMA-PET/CT scans were done for treatment response evaluation. ^18^ F-FDG-PET/CT scan showed reduction in the FDG avid disease bulk of left lung ( Fig. 1B ), but there were multiple new-onset FDG avid lesions like parenchymal lung nodules, a hypodense lesion in segment VI of liver ( Fig. 2A–C ) and a metastasis in right temporal lobe ( Fig. 2D, 2E ). ^68^ Ga-PSMA-11 PET/CT scan at this point showed near complete resolution of all the previously seen PSMA expressing primary and metastatic lesions, but a new-onset PSMA expressing lesion in liver segment VI ( Fig. 1D ). Serum PSA had reduced to 0.11 ng/mL. This liver lesion showed moderate FDG as well as PSMA avidity. Biopsy of the liver lesion showed metastatic small cell carcinoma ( Fig. 3A ). The immunohistochemistry was strongly positive for synaptophysin ( Fig. 3B ), chromogranin ( Fig. 3C ), and TTF-1 ( Fig. 3D ), patchy positive for AE1/AE3 ( Fig. 3E ), and negative for NKX3.1 ( Fig. 3F ).

## Discussion

Metachronous malignancies pose unique diagnostic and therapeutic challenges in the management. Ceritinib, an ALK inhibitor, has shown positive results in ALK+ lung adenocarcinoma in ASCEND-4 and ASCEND-5 trials. 13 14 As illustrated in our case, lung adenocarcinoma showed excellent reduction of disease burden at the primary site following this targeted therapy but new-onset pulmonary, brain, and hepatic metastases had developed. Prostate adenocarcinoma of neuroendocrine differentiation can also express ALK positivity in approximately 9% cases. 15 The prostatic adenocarcinoma in this case had shown near-complete response with significant reduction of serum PSA, except for the new-onset moderately PSMA avid hepatic metastasis. Serum PSA levels can be low in small cell differentiated prostate carcinoma. 16 Prostate adenocarcinoma of neuroendocrine differentiation usually retains the increased PSMA expression and are imaged with ^68^ Ga-PSMA PET/CT. 17 The hepatic metastases showed FDG as well as PSMA avidity and posed an uncertainty about the malignancy, which has metastasized at this location. The histopathology of this lesion came out to be of small cell type, which raised the uncertainty further as both of these malignancies can undergo such transformation.

Immunohistochemistry revealed TTF-1 positivity and NKX3.1 negativity. Although staining for NKX3.1 protein is positive in the majority of primary prostatic adenocarcinomas, it has been shown to be downregulated in many high-grade prostate cancers, and completely lost in the majority of metastatic prostate cancers (65–78% of lesions). 18 TTF-1 can be positive in half of the small cell carcinoma of prostate and small cell neuroendocrine prostate carcinoma arising from conventional prostate adenocarcinoma treated with androgen deprivation therapy. 19 20 In our case, the liver lesion had shown NKX3.1 negativity and TTF-1 positivity, which is consistent with the evidences of literature.

Brain metastases are common in patients with ALK+ metastatic nonsmall cell lung cancer. In one retrospective study, out of 1,040 patients with ALK+ NSCLC treated with second-generation ALK inhibitors as first-line therapy, brain metastases were found in 28% cases at baseline itself, whereas in 751 patients without baseline brain metastases, the cumulative incidence of the same was 20% after 5 years. 21 In our case, the patient was similarly treated with second-generation ALK inhibitor ceritinib as the first-line therapy. The new-onset brain metastases of right temporal lobe showed significant FDG uptake (hypermetabolism) but did not show PSMA expression, which was likely the metastases from primary ALK+ NSCLC.

These immunohistochemistry findings, presence of FDG-avid, PSMA nonavid brain metastasis, and FDG as well as PSMA avid liver metastasis led to the conclusion that the new-onset brain metastasis had originated from lung adenocarcinoma, whereas the new-onset liver lesion of small cell histology was the result of metastasis from small cell differentiation of prostate adenocarcinoma of neuroendocrine differentiation.

## Conclusion

The present case highlights the significance of correlating multiple diagnostic modalities, in this case dual-tracer PET/CT and histopathology with immunohistochemistry and tumor marker biochemistry to achieve accurate diagnoses and select suitable treatments in case of metachronous malignancies, particularly when the two malignancies can have different metastatic site preference and require markedly different standard therapies.
